# Clinical Relevance of Vitamins and Carotenoids With Liver Steatosis and Fibrosis Detected by Transient Elastography in Adults

**DOI:** 10.3389/fnut.2021.760985

**Published:** 2021-11-11

**Authors:** Xiaohui Liu, Hong Shen, Mingfeng Chen, Jun Shao

**Affiliations:** Department of Ultrasound Diagnosis, The First People's Hospital of Kunshan, Kunshan Hospital Affiliated to Jiangsu University, Kunshan, China

**Keywords:** carotenoids, liver fibrosis, liver steatosis, dose-response analysis, vitamins, national health and nutrition examination survey

## Abstract

**Background:** Vitamins and carotenoids may be involved in the pathogenesis of non-alcoholic fatty liver disease (NAFLD). Previously related publications mainly focused on vitamin D and vitamin E, and studies on other vitamins and carotenoids and NAFLD are scarce.

**Methods:** This study aimed to explore the clinical relevance of vitamin A, B vitamins (vitamin B1, vitamin B2, niacin, vitamin B6, folate, vitamin B12, and choline), vitamin C and carotenoids (α-carotene, β-carotene, β-cryptoxanthin, lycopene, lutein + zeaxanthin) with liver steatosis and fibrosis in the 2017–2018 NHANES (*N* = 4,352). Liver steatosis and fibrosis were detected by transient elastography. Logistic regression, linear regression and restricted cubic splines were adopted to explore the non-linear dose-response relationships.

**Results:** Higher intakes of vitamin C [0.68 (0.50–0.93)] and β-carotene [0.71 (0.54–0.93)] were inversely associated with liver steatosis. Higher levels of serum vitamin C [0.45 (0.32–0.62)] were inversely associated with liver fibrosis, while higher intakes of choline [1.43 (1.04–1.98)] and α-carotene [1.67 (1.01–2.74)] were positively associated with liver fibrosis. In addition, marginally inverse association between lutein + zeaxanthin and liver steatosis and positive association between vitamin B12 and liver fibrosis were found. In linear regression, the above-mentioned associations between vitamin C, β-carotene, and lutein + zeaxanthin and liver steatosis, and serum vitamin C, choline, α-carotene, and vitamin B12 and liver fibrosis were also found. The above-mentioned associations were mainly linear, while the relationship between β-carotene and liver steatosis might be non-linear.

**Conclusion:** Vitamin C, α-carotene, β-carotene, lutein + zeaxanthin, choline and vitamin B12 may be associated with liver steatosis and fibrosis.

## Introduction

Non-alcoholic fatty liver disease (NAFLD) is a major cause of liver disease worldwide, and 25% of the adult population in the world has NAFLD (1). NAFLD ranges from simple steatosis to non-alcoholic steatohepatitis with or without hepatic fibrosis in the absence of excessive alcohol intake (2). Although substantial advances have been made in NAFLD mechanisms, diagnostics, and treatment (2), NAFLD remains the center of attention within liver owing to its high prevalence and growing burden in the general population (3). The most effective type of long-term treatment of NAFLD remains lifestyle modification (4, 5). Although the pathogenesis of NAFLD is not well-understood, the leading hypothesis is the hepatic fat accumulation owing to insulin resistance followed by oxidative stress arising from reactive oxygen species produced primarily by mitochondria (6, 7). The liver plays a critical role in the metabolism of vitamins, and deregulation of vitamins metabolism may contribute to the initiation and progression of NAFLD (6–8). The evidence available supports the potential benefits of vitamin D and vitamin E on NAFLD development (9, 10). However, studies on other vitamins and carotenoids and NAFLD are scarce. Transient elastography is considered the non-invasive standard tool for assessing liver fibrosis (11), and provides higher sensitivity and specificity for detecting liver steatosis than liver enzymes (12), and has been adopted to detect liver steatosis and liver fibrosis in the general population (13, 14). Therefore, based on the 2017–2018 National Health and Nutrition Examination Survey (NHANES), we aimed to examine the clinical relevance of vitamin A, B vitamins (vitamin B1, vitamin B2, niacin, vitamin B6, folate, vitamin B12, and choline), vitamin C and carotenoids (α-carotene, β-carotene, β-cryptoxanthin, lycopene, lutein + zeaxanthin) with liver steatosis and fibrosis detected by transient elastography in adults.

## Materials and Methods

### Eligible Sample

NHANES is a national population-based survey program assessing the health and nutritional status of the civilian non-institutionalized general U.S. population. The NHANES data are released every 2 years. Data from the 2017–2018 NHANES cycle were used in this analysis, because this cycle specifically provided data for liver steatosis and fibrosis.

All participants aged 20 years and over were eligible. Participants were excluded if (1) their dietary intakes of vitamins, carotenoids, or serum vitamin C levels are missing; (2) they are infected with hepatitis B or C (defined by the presence of hepatitis C antibodies or the presence of hepatitis B surface antigen); (3) they have significant alcohol consumption (>30 g/day in men and >20 g/day in women) (13); and (4) their data of transient elastography are missing. The protocol was approved by the National Center for Health Statistics Institutional Review Board, and all subjects provided written informed consent.

### Vitamins and Carotenoids

Dietary intakes of vitamin A, B vitamins (vitamin B1, vitamin B2, niacin, vitamin B6, folate, vitamin B12, and choline), vitamin C and carotenoids (α-carotene, β-carotene, β-cryptoxanthin, lycopene, lutein + zeaxanthin) were included as exposures. All NHANES participants are eligible for two 24-h dietary recall interviews. The first dietary recall interview is collected in-person in the Mobile Examination Center and the second interview is collected by telephone 3–10 days later. In this analysis, dietary intakes of vitamin and carotenoids were assessed using 1-day values for individuals with single recalls and 2-day means for others.

Serum vitamin C concentrations are also available in the dataset, and thus were also included in this analysis. Vitamin C in serum is measured using isocratic ultra-high performance liquid chromatography. Serum specimens were processed, stored, and shipped to the Division of Laboratory Sciences, National Center for Environmental Health, Centers for Disease Control and Prevention, Atlanta, GA for analysis. Vials are stored under appropriate frozen (−30°C) conditions until they are shipped to National Center for Environmental Health for testing. The NHANES quality assurance and quality control protocols meet the 1988 Clinical Laboratory Improvement Act mandates.

### Transient Elastography

The main goals of the transient elastography component are to provide objective measures for two important liver disease manifestations: liver fibrosis and liver steatosis. Liver fibrosis was measured by FibroScan® which uses ultrasound and the vibration controlled transient elastography to derive liver stiffness. The device also simultaneously measures the ultrasound attenuation related to the presence of liver steatosis and records the controlled attenuation parameter as the indicator for the fatness in the liver. The elastography exam was performed by NHANES health technicians, who were trained and certified by NHANES staff and the equipment manufacturer. A detailed description of quality assurance and quality control measures considered for this component can be found in the Procedures Manual (15). We defined liver steatosis as controlled attenuation parameter scores of 263 dB/m or more and liver stiffness values of 8 kPa or higher were considered to have significant fibrosis (11, 16, 17).

### Covariates

According to the previously related publication (13), we included the following covariates that are risk factors of NAFLD: age (in 10-year increments), sex, race/ethnicity (Mexican–American, Other Hispanic, Non-hispanic White, Non-hispanic Black, Other Races), education (≤ high school, >high school), recreational physical activity (at least 10 min continuously per week), smoking (current smoker, former smoker, never smoker), alcohol drinking (continuous), hypertension, diabetes, body mass index (under/normal weight: <25 kg/m^2^, overweight: 25– <30 kg/m^2^, obesity: ≥30 kg/m^2^), and dietary intakes of cholesterol. Diabetes was defined using a previous diagnosis of diabetes or taking diabetic pills to lower blood sugar or, if diabetes was not previously diagnosed, by a hemoglobin A1c level ≥ 6.5%, or a fasting plasma glucose level ≥ 126 mg/dL (18). Subjects were considered hypertensive if they were taking antihypertensive medication, if their systolic blood pressure exceeded 130 mmHg, or if their mean diastolic blood pressure exceeded 80 mmHg (mean values of three measurements) (19).

### Statistical Analysis

Logistic regression was adopted to explore the associations between vitamins and carotenoids and liver steatosis and fibrosis. Subjects were classified into tertiles according to their dietary intakes of vitamins, carotenoids or serum vitamin C concentrations, and the odds ratios (ORs) and 95% confidence intervals (CIs) of liver steatosis and fibrosis for subjects in tertile 2 and tertile 3 were calculated as compared to those in tertile 1. There are three different logistic regression models. Model 1 was adjusted for age, sex and race/ethnicity. Model 2 was adjusted for covariates in model 1, and also education, physical activity, smoking and alcohol drinking. Model 3 was adjusted for covariates in model 2, and also body mass index, hypertension, diabetes, and dietary intakes of cholesterol. The potential non-linear dose-response relationships between vitamins, carotenoids, and liver steatosis and fibrosis were examined by modeling dietary intakes of vitamins, carotenoids, or serum vitamin C concentrations using restricted cubic splines with 3 knots at percentiles 25, 50, and 75 of the distribution (20). A *P*-value for non-linearity was calculated by testing the null hypothesis that the coefficient of the second spline is equal to 0 (20). We also conducted multivariable linear regression model with the outcomes and exposures expressed as continuous variables. In sensitivity analysis, we explored the associations between vitamin intakes from both diets and supplements and liver steatosis and liver fibrosis. All analyses were conducted using STATA version 12.0, and *P* ≤ 0.05 was considered statistically significant. Appropriate strata, cluster, and weights were considered in all analyses as suggested by NHANES.

## Results

### Population Characteristics

According to the exclusion criteria, individuals who are infected with hepatitis B or C (*N* = 79), who they have significant alcohol consumption (*N* = 457) and whose data of transient elastography are missing (*N* = 630), a total of 4,352 subjects were potentially eligible for this analysis. Among the 4,352 subjects, 4,116 provided data of serum vitamin C concentrations and 3,940 provided dietary intakes of vitamins and carotenoids. The weighted prevalence of was 56.47% for liver steatosis and 15.05% for liver fibrosis, respectively. Patients with liver steatosis and fibrosis showed higher prevalence of diabetes, obesity and hypertension (all *P* < 0.01). Patients with liver fibrosis and liver steatosis had higher dietary intakes of vitamin B12, choline and cholesterol, while had lower dietary intakes of vitamin C and lutein + zeaxanthin and lower levels of serum vitamin C concentrations (all *P* ≤ 0.05; Table 1).

**Table 1:** Population characteristics by presence of liver steatosis and liver fibrosis.

**Characteristics**	**Overall**	**Liver steatosis**			**Liver fibrosis**		
		**Yes**	**No**	***P* ^**a**^**	**Yes**	**No**	***P* ^**a**^**
Age, year	51.49 (17.56)	53.74 (16.29)	48.27 (18.76)	<0.01	55.64 (15.84)	50.67 (17.76)	<0.01
Men, %	48.20	51.72	43.16	<0.01	56.37	46.63	<0.01
Diabetes, %	21.95	30.51	9.72	<0.01	39.94	18.46	<0.01
Obesity, %	42.12	57.89	19.59	<0.01	69.35	36.90	<0.01
Hypertension, %	59.89	68.28	47.91	<0.01	73.23	57.30	<0.01
Education, %				0.14			0.01
≤ High school	44.26	45.18	42.93		48.58	43.40	
>High school	55.74	54.82	57.07		51.42	56.60	
Race/Hispanic origin (%)				<0.01			<0.01
Mexican American	13.74	16.52	9.77		15.86	13.33	
Other hispanic	9.52	10.16	8.60		9.07	9.60	
Non-hispanic white	33.44	33.95	32.72		37.11	32.72	
Non-hispanic black	23.47	19.96	28.48		24.93	23.20	
Other race	19.83	19.41	20.44		13.03	21.15	
Smoking status (%)				<0.01			<0.01
Current smoker	17.10	15.23	19.77		16.86	17.14	
Former smoker	23.53	26.68	19.04		29.75	22.33	
Never smoker	59.37	58.09	61.19		53.40	60.53	
Physical activity^a^				<0.01			<0.01
Yes	60.24	56.73	62.70		59.30	65.01	
No	39.76	43.27	37.30		40.70	34.99	
**Dietary intakes of**							
Vitamin A, RAE (μg/d)	605.65 (502.34)	611.35 (539.20)	597.51 (444.45)	0.40	621.65 (605.21)	602.61 (479.99)	0.38
Vitamin B1 (mg/d)	1.54 (0.79)	1.56 (0.78)	1.52 (0.80)	0.16	1.59 (0.82)	1.53 (0.78)	0.07
Vitamin B2 (mg/d)	1.90 (1.13)	1.91 (1.08)	1.91 (1.20)	0.97	1.92 (1.08)	1.90 (1.14)	0.71
Vitamin B6 (mg/d)	2.02 (1.54)	2.03 (1.49)	2.00 (1.61)	0.60	2.01 (1.31)	2.02 (1.58)	0.89
Vitamin B12 (μg/d)	4.48 (4.18)	4.59 (4.29)	4.32 (4.01)	0.04	4.76 (4.54)	4.42 (4.10)	0.05
Choline (mg/d)	314.62 (159.40)	319.74 (158.42)	307.31 (160.56)	0.02	327.06 (158.99)	312.20 (159.37)	0.03
Niacin (mg/d)	24.25 (13.87)	24.32 (13.27)	24.14 (14.68)	0.68	24.47 (12.63)	24.20 (14.09)	0.66
Folate, DFE (μg/d)	483.42 (313.37)	482.65 (297.15)	484.53 (335.27)	0.85	498.94 (397.87)	480.48 (294.27)	0.17
Vitamin C (mg/d)	79.22 (78.39)	76.14 (74.58)	83.63 (83.36)	<0.01	75.90 (72.30)	79.86 (79.49)	0.24
Alpha-Carotene (μg/d)	396.24 (1,154.66)	413.04 (1,366.03)	372.25 (756.23)	0.28	436.00 (1,979.85)	388.47 (914.15)	0.34
Beta-Carotene (μg/d)	2,400.22 (4,044.30)	2,360.12 (4,339.64)	2,457.50 (3,580.95)	0.46	2,365.69 (4,969.46)	2,406.28 (3,841.10)	0.82
Beta-Cryptoxanthin (μg/d)	97.80 (251.64)	98.80 (256.48)	96.37 (244.62)	0.77	111.74 (332.81)	95.09 (232.72)	0.13
Lutein + zeaxanthin (μg/d)	1,666.57 (2,936.97)	1,548.67 (2,734.24)	1,834.98 (3,197.73)	<0.01	1,578.19 (2,609.11)	1,683.20 (2,995.67)	0.41
Lycopene (μg/d)	4,498.08 (6,755.69)	4,482.38 (6,645.59)	4,520.50 (6,911.93)	0.86	4,329.38 (6,167.15)	4,531.66 (6,862.87)	0.49
Alcohol (g/d)	2.65 (5.74)	2.58 (5.72)	2.76 (5.76)	0.31	2.63 (5.65)	2.66 (5.77)	0.88
Cholesterol (mg/d)	298.25 (208.27)	304.15 (208.15)	289.82 (208.22)	0.03	319.36 (226.25)	294.17 (204.38)	<0.01
Serum vitamin C (mg/dL)	0.80 (0.49)	0.85 (0.48)	0.97 (0.51)	<0.01	0.76 (0.48)	0.92 (0.49)	<0.01

*Values are means ± SDs for continuous variables.*

*^a^ANOVA test was performed for continuous variables, and Chi-square test was performed for categorical variables.*

*RAE, retinol activity equivalents; DFE, dietary folate equivalents*.

### Associations With Liver Steatosis

Overall, the findings between vitamins and carotenoids and liver steatosis were similar between model 1 and model 2. In model 2, comparing the highest to lowest tertile, higher dietary intakes of vitamin C [OR (95% CI): 0.59 (0.42–0.81)] and β-carotene [0.70 (0.57–0.87)] and serum vitamin C concentrations [0.50 (0.38–0.66)] were inversely associated with liver steatosis, respectively. However, after further adjustment for obesity, diabetes, hypertension and cholesterol intakes (model 3), only dietary intakes of vitamin C [0.68 (0.50–0.93)] and β-carotene [0.71 (0.54–0.93)] were significantly associated with liver steatosis. In addition, a marginally inverse association between lutein + zeaxanthin and liver steatosis were found [0.74 (0.53–1.04)]. No associations were found between other vitamins and carotenoids and liver steatosis in model 3 (Table 2). The findings from multivariable linear regression model are consistent with those from the logistic regression, and the above-mentioned associations between vitamin C, β-carotene, and lutein + zeaxanthin and liver steatosis were also observed (Supplementary Table 2). Similar findings were also found in sensitivity analysis including intakes from Supplementary Table 3.

**Table 2:** Associations between vitamins and carotenoids and liver steatosis.

**Exposure**	**Model 1**		**Model 2**		**Model 3**	
	**T2 vs. T1**	**T3 vs. T1**	**T2 vs. T1**	**T3 vs. T1**	**T2 vs. T1**	**T3 vs. T1**
	**OR (95% CI)**	**OR (95% CI)**	**OR (95% CI)**	**OR (95% CI)**	**OR (95% CI)**	**OR (95% CI)**
Vitamin A, RAE	1.00 (0.78–1.27)	0.76 (0.53–1.10)	1.02 (0.78–1.32)	0.80 (0.56–1.15)	1.05 (0.77–1.44)	0.83 (0.55–1.25)
Vitamin B1	1.02 (0.83–1.25)	1.02 (0.77–1.36)	1.03 (0.85–1.25)	1.05 (0.80–1.38)	1.09 (0.85–1.39)	1.20 (0.86–1.68)
Vitamin B2	0.93 (0.70–1.23)	0.88 (0.60–1.28)	0.95 (0.71–1.28)	0.92 (0.63–1.33)	1.08 (0.76–1.52)	1.03 (0.65–1.62)
Vitamin B6	1.02 (0.78–1.31)	0.86 (0.55–1.35)	1.02 (0.77–1.34)	0.89 (0.57–1.40)	1.08 (0.86–1.35)	0.91 (0.55–1.50)
Vitamin B12	1.11 (0.77–1.61)	1.06 (0.70–1.62)	1.14 (0.78–1.67)	1.09 (0.72–1.64)	1.09 (0.69–1.73)	1.07 (0.72–1.58)
Choline	1.10 (0.80–1.52)	1.06 (0.76–1.49)	1.12 (0.82–1.55)	1.13 (0.81–1.59)	1.22 (0.81–1.81)	1.23 (0.67–2.25)
Niacin	1.09 (0.74–1.61)	1.15 (0.75–1.75)	1.10 (0.75–1.62)	1.17 (0.77–1.77)	1.25 (0.89–1.76)	1.22 (0.77–1.92)
Folate, DFE	0.94 (0.68–1.29)	0.93 (0.70–1.24)	0.96 (0.69–1.32)	0.96 (0.72–1.28)	1.01 (0.75–1.36)	1.06 (0.74–1.51)
Vitamin C	0.71 (0.53–0.95)*	0.56 (0.41–0.77)**	0.72 (0.54–0.97)*	0.59 (0.42–0.81)**	0.75 (0.56–0.99)*	0.68 (0.50–0.93)*
α-carotene	1.06 (0.76–1.48)	0.89 (0.68–1.16)	1.07 (0.79–1.47)	0.93 (0.69–1.25)	1.13 (0.77–1.67)	1.08 (0.80–1.45)
β-carotene	0.92 (0.70–1.22)	0.67 (0.54–0.83)**	0.95 (0.72–1.26)	0.70 (0.57–0.87)**	0.90 (0.69–1.17)	0.71 (0.54–0.93)*
β-cryptoxanthin	1.05 (0.80–1.37)	0.84 (0.65–1.09)	1.09 (0.80–1.48)	0.86 (0.67–1.12)	1.12 (0.81–1.54)	0.82 (0.57–1.18)
Serum vitamin C	0.71 (0.52–0.96)*	0.51 (0.39–0.66)**	0.73 (0.52–1.01)	0.50 (0.38–0.66)**	0.81 (0.58–1.13)	0.82 (0.61–1.10)
Lutein + zeaxanthin	0.90 (0.69–1.16)	0.74 (0.55–1.00)*	0.90 (0.70–1.16)	0.79 (0.58–1.08)	0.75 (0.51–1.09)	0.74 (0.53–1.04)
Lycopene	1.08 (0.83–1.40)	1.00 (0.81–1.23)	1.10 (0.85–1.42)	1.03 (0.84–1.26)	1.25 (0.90–1.73)	1.05 (0.79–1.41)

*T1, tertile 1; T2, tertile 2; T3, tertile 3.*

*OR (95% CI), odds ratio (95% confidence interval).*

*P < 0.05, **P < 0.01.

*RAE, retinol activity equivalents; DFE, dietary folate equivalents.*

*Model 1 was adjusted for age, sex, and race/ethnicity.*

*Model 2 was adjusted for covariates in model 1, and also education, physical activity, smoking, and alcohol drinking.*

*Model 3 was adjusted for covariates in model 2, and also body mass index, hypertension, diabetes, and dietary intakes of cholesterol*.

### Associations With Liver Fibrosis

Overall, the findings between vitamins and carotenoids and liver fibrosis were similar between model 1 and model 2. In model 2, comparing the highest to lowest tertile, higher dietary intakes of vitamin B12 [1.49 (1.01–2.18)] and choline [1.56 (1.11–2.21)] were positively associated with liver fibrosis. In addition, higher dietary intakes of α-carotene [tertile 2 vs. tertile 1: 1.85 (1.16–2.95)] were also positively associated with liver fibrosis. Higher levels of serum vitamin C were inversely associated with liver fibrosis [0.45 (0.29–0.70)]. After further adjustment for obesity, diabetes, hypertension and cholesterol intakes (model 3), higher dietary intakes of choline [tertile 2 vs. tertile 1: 1.43 (1.04–1.98)] were positively associated with liver fibrosis. Higher levels of serum vitamin C were inversely associated with liver fibrosis in model 3 [tertile 2 vs. tertile 1: 0.45 (0.32–0.62)].

No associations were found between other vitamins and carotenoids and liver fibrosis in model 3 (Table 3). The findings from multivariable linear regression model are consistent with those from the logistic regression, and the above-mentioned associations between serum vitamin C, choline, α-carotene and vitamin B12 and liver fibrosis were also observed (Supplementary Table 2). Similar findings were also found in sensitivity analysis including intakes from Supplementary Table 4.

**Table 3:** Associations between vitamins and carotenoids and liver fibrosis.

**Exposures**	**Model 1**		**Model 2**		**Model 3**	
	**T2 vs. T1**	**T3 vs. T1**	**T2 vs. T1**	**T3 vs. T1**	**T2 vs. T1**	**T3 vs. T1**
	**OR (95% CI)**	**OR (95% CI)**	**OR (95% CI)**	**OR (95% CI)**	**OR (95% CI)**	**OR (95% CI)**
Vitamin A, RAE	1.12 (0.69–1.83)	1.18 (0.71–1.94)	1.20 (0.75–1.91)	1.30 (0.80–2.09)	1.12 (0.73–1.71)	1.18 (0.72–1.93)
Vitamin B1	0.98 (0.71–1.36)	1.27 (0.78–2.08)	0.99 (0.73–1.35)	1.34 (0.84–2.12)	0.98 (0.72–1.32)	1.31 (0.80–2.15)
Vitamin B2	1.06 (0.75–1.50)	1.00 (0.65–1.54)	1.10 (0.78–1.54)	1.07 (0.71–1.61)	1.10 (0.73–1.66)	0.92 (0.59–1.44)
Vitamin B6	1.11 (0.72–1.72)	1.10 (0.68–1.76)	1.15 (0.78–1.70)	1.17 (0.75–1.85)	1.10 (0.78–1.57)	1.07 (0.65–1.77)
Vitamin B12	1.53 (1.03–2.29)*	1.44 (0.97–2.13)	1.59 (1.08–2.33)*	1.49 (1.01–2.18)*	1.40 (0.94–2.09)	1.23 (0.76–2.01)
Choline	1.49 (1.04–2.14)*	1.45 (1.00–2.10)*	1.57 (1.13–2.19)*	1.56 (1.11–2.21)*	1.43 (1.04–1.98)*	1.01 (0.62–1.64)
Niacin	0.95 (0.54–1.66)	1.23 (0.80–1.89)	0.96 (0.56–1.66)	1.29 (0.86–1.91)	0.98 (0.55–1.76)	1.12 (0.74–1.70)
Folate, DFE	0.80 (0.43–1.48)	0.95 (0.57–1.58)	0.82 (0.45–1.48)	1.00 (0.62–1.62)	0.82 (0.47–1.41)	1.04 (0.63–1.72)
Vitamin C	0.81 (0.55–1.19)	0.96 (0.63–1.48)	0.84 (0.56–1.26)	1.05 (0.71–1.55)	0.82 (0.51–1.32)	1.18 (0.79–1.76)
α-carotene	1.74 (1.10–2.75)*	1.34 (0.85–2.11)	1.85 (1.16–2.95)*	1.48 (0.94–2.33)	2.02 (1.37–2.99)**	1.67 (1.01–2.74)*
β-carotene	1.00 (0.66–1.52)	1.10 (0.61–1.99)	1.07 (0.69–1.66)	1.22 (0.66–2.27)	1.12 (0.70–1.81)	1.28 (0.65–2.54)
β-cryptoxanthin	0.88 (0.55–1.42)	1.24 (0.78–1.99)	0.90 (0.55–1.47)	1.29 (0.80–2.09)	0.84 (0.51–1.40)	1.16 (0.63–2.14)
Serum vitamin C	0.42 (0.32–0.56)**	0.44 (0.29–0.67)**	0.43 (0.32–0.57)**	0.45 (0.29–0.70)**	0.45 (0.32–0.62)**	0.62 (0.36–1.08)
Lutein + zeaxanthin	1.26 (0.84–1.90)	1.09 (0.67–1.76)	1.30 (0.88–1.93)	1.22 (0.75–1.97)	1.03 (0.66–1.62)	1.11 (0.61–2.02)
Lycopene	0.69 (0.41–1.16)	0.85 (0.46–1.57)	0.70 (0.42–1.17)	0.89 (0.49–1.60)	0.71 (0.42–1.21)	0.88 (0.48–1.63)

*T1, tertile 1; T2, tertile 2; T3, tertile 3.*

*OR (95% CI), odds ratio (95% confidence interval).*

*P < 0.05, **P < 0.01.

*RAE, retinol activity equivalents; DFE, dietary folate equivalents.*

*Model 1 was adjusted for age, sex, and race/ethnicity.*

*Model 2 was adjusted for covariates in model 1, and also education, physical activity, smoking, and alcohol drinking.*

*Model 3 was adjusted for covariates in model 2, and also body mass index, hypertension, diabetes, and dietary intakes of cholesterol*.

### Dose-Response Relationships

The median values in the first category (tertile 1) of exposures were used as the references values in dose-response analysis. Dose-response analysis with restricted cubic splines showed that the departure from a linear relationship was not significant between vitamin C (P_fornon−linearity_ = 0.30) and lutein + zeaxanthin (P_fornon−linearity_ = 0.75) and liver steatosis, while the departure from a linear relationship was significant between β-carotene (P_fornon−linearity_ = 0.02) and liver steatosis. The departure from a linear relationship was not significant between serum vitamin C (P_fornon−linearity_ = 0.34), choline (P_fornon−linearity_ = 0.36), α-carotene (P_fornon−linearity_ = 0.36) and vitamin B12 (0.45) and liver fibrosis.

## Discussion

Results from this national population-based survey showed that vitamin C, α-carotene, β-carotene, lutein + zeaxanthin, choline, and vitamin B12 may be associated with liver steatosis and fibrosis. Dose-response analysis showed that the above-mentioned associations were mainly linear, while the evidence of non-linear relationship between β-carotene and liver steatosis were observed.

Oxidative stress has been reported to be causative in NAFLD initiation and progression (6, 7). Day and James proposed that NAFLD requires a double hit: the first hit produces steatosis by a high-fat diet or diabetes, and then the second is a source of oxidative stress capable of initiating significant lipid peroxidation (21). Vitamin C is a powerful antioxidant in human health by scavenging free radicals, which could protect hepatic cells from lipotoxicity-induced cellular oxidative stress (8, 22). In addition, vitamin C is also believed to play a role in circulating and hepatic lipid homeostasis (22). Observational studies on vitamin C and NAFLD are limited. A cross-sectional study in Brazil found a high proportion of inadequate serum vitamin C (27%) in 72 NAFLD patients (23). Vitamin C intakes were also found lower in NAFLD patients (*N* = 200) than the healthy controls (*N* = 400) in Iran (24), while no significant difference was found in other case-control studies [120 Jordanian adults (25), 317 Iranians (26), 101 Canadians (27), 52 Indians (28)]. Therefore, previous publications on vitamin C and NAFLD usually included small number of participants. Metabolic comorbidities associated with NAFLD included obesity, type 2 diabetes, hyperlipidemia, hypertension, and metabolic syndrome (1). Vitamin C could improve lipid profile (29), glycemic control and cardiovascular risk factors including hypertension (30, 31). A recommended dietary allowance (RDA) of 90 mg/day for adult men and 75 mg/day for adult women is set to provide antioxidant protection (32). Therefore, whether the effect of vitamin C on liver fibrosis is more evident than that on liver steatosis should be confirmed further.

Vitamin B1 functions as a coenzyme in the metabolism of carbohydrates and branched-chain amino acids, and the RDA for adults is 1.2 mg/day for men and 1.1 mg/day for women (33). Vitamin B12 functions as a coenzyme for a critical methyl transfer reaction, and the RDA for adults is 2.4 μg/day of vitamin B12 (33). The potential mechanisms of vitamin B1 and vitamin B12 in NAFLD remains unclear, and findings from clinical studies are scarce (8). Higher serum levels of vitamin B12 were positively correlated with the severity of steatosis and fibrosis in 614 Brazilian patients (34). Dietary intakes of vitamin B1 and vitamin B12 were also found higher in patients with NAFLD patients than controls in 120 adult Jordanians (25) and 101 Canadians (27). However, the differences of dietary intakes of vitamin B1 and vitamin B12 in 317 Iranians (26) and serum vitamin B12 concentrations in 54 participants in Greece (35) were not significant between with NAFLD patients and controls. In contrast, serum vitamin B12 concentrations were lower in NAFLD patients and controls in 75 Turks (36), and low vitamin B12 levels were significantly associated with a higher fibrosis grade and non-alcoholic steatohepatitis activity in 83 patients in Israel (37). Therefore, previous publications on vitamin B1 and B12 and NAFLD also usually included small number of participants and did not adjust for covariates in univariate analysis. Our results are consistent with those from several publications in which vitamin B1 and B12 levels were higher in NAFLD patients than controls. In addition, higher vitamin B12 levels were associated with a greater risk of total mortality and combined fatal and non-fatal coronary events in prospective cohort studies (38).

Putative mechanisms of other vitamins and carotenoids in NAFLD pathogenesis have been summarized elsewhere (8), and these mechanisms include antioxidant, antifibrotic, immune effects, and lipoprotective effects (8). However, dietary intakes of vitamin A were not associated with liver steatosis and liver fibrosis in this analysis, and the inverse association between β-carotene and liver steatosis was not significant after further adjustment for metabolic comorbidities associated with NAFLD including obesity, diabetes, and hypertension. Previous studies on vitamin A (23–27, 39) and β-carotene (23, 26) are also limited and not consistent. As shown above, previous studies usually included small number of participants and did not adjust for other covariates.

To our knowledge, this is the first national population-based survey to explore the associations between these vitamins and carotenoids and liver fibrosis and steatosis. We also explored the potential non-linear dose-response relationships and considered a number of covariates. However, there are also limitations within this study. First, this is a cross-sectional study and we cannot determine the causality, which should be confirmed by prospective studies. In addition, patients may change their dietary habits after diagnosis of NAFLD. However, studies on these vitamins and carotenoids are limited, and there are no clinical guidelines to date recommending or limiting these vitamins and carotenoids intake for NAFLD prevention. Second, misclassification of these vitamins and carotenoids consumption could be of concern; however, non-differential misclassification at baseline should have weakened the association. Third, although we considered a number of covariates, the extent to which they were adjusted and residual confounding by other unmeasured factors are also of concern in observational studies. However, the findings were similar between model 1 and model 2, although several associations were not significant after further adjustment for obesity, diabetes, and hypertension. Finally, a number of participants were excluded from this analysis. In a recent study using data from the 2017–2018 NHANES cycle, the prevalence of non-alcoholic fatty liver disease (controlled attenuation parameter scores of ≥248 dB/m) was 56.7%, and the prevalence of significant liver fibrosis (≥7.9 kPa) was 14.5% (14), which are comparable to the findings in this study.

In summary, results from this national population-based survey showed that vitamin C, α-carotene, β-carotene, lutein + zeaxanthin, choline, and vitamin B12 may be associated with liver steatosis and fibrosis. The above-mentioned associations were mainly linear, while the relationship between β-carotene and liver steatosis might be non-linear. These findings need to be confirmed by prospective studies.

## Data Availability Statement

The datasets generated during and/or analyzed during the current study are available in the NHANES: https://www.cdc.gov/nchs/nhanes/.

## Ethics Statement

NHANES was approved by the National Center for Health Statistics Research Ethics Review Board. Ethical approval for this study is deemed exempt because this study uses publically available secondary data. The patients/participants provided their written informed consent to participate in this study.

## Author Contributions

XL and JS: conceptualization. XL: methodology and resources. XL and HS: formal analysis. XL, HS, and MC: writing. JS: supervision. All authors contributed to the article and approved the submitted version.

## Conflict of Interest

The authors declare that the research was conducted in the absence of any commercial or financial relationships that could be construed as a potential conflict of interest.

## Publisher's Note

All claims expressed in this article are solely those of the authors and do not necessarily represent those of their affiliated organizations, or those of the publisher, the editors and the reviewers. Any product that may be evaluated in this article, or claim that may be made by its manufacturer, is not guaranteed or endorsed by the publisher.
