# Supplementary material for: Clinical Relevance of Vitamins and Carotenoids With Liver Steatosis and Fibrosis Detected by Transient Elastography in Adults
Source: Front Nutr. 2021 Nov 11;8:760985. doi: 10.3389/fnut.2021.760985 (PMC8632634; doi:10.3389/fnut.2021.760985)
Supplement: Supplementary file 1 [file Data_Sheet_1.docx]

Supplementary Table 1 STROBE statement checklist of items that should be included in reports of observational studies

|  | **Page** | **Item No** | **Recommendation** |
| --- | --- | --- | --- |
| **Title and abstract** |  |  |  |
|  | 1  2-3 | 1 | (*a*) Indicate the study's design with a commonly used term in the title or the abstract |
|  |  |  | (*b*) Provide in the abstract an informative and balanced summary of what was done and what was found |
| **Introduction** |  |  |  |
| Background/rationale | 4 | 2 | Explain the scientific background and rationale for the investigation being reported |
| Objectives | 4 | 3 | State specific objectives, including any prespecified hypotheses |
| **Methods** |  |  |  |
| Study design | 5 | 4 | Present key elements of study design early in the paper |
| Setting | 5 | 5 | Describe the setting, locations, and relevant dates, including periods of recruitment, exposure, follow-up, and data collection |
| Participants | 5 | 6 | (*a*) *Cohort study*?Give the eligibility criteria, and the sources and methods of selection of participants. Describe methods of follow-up*Case-control study*?Give the eligibility criteria, and the sources and methods of case ascertainment and control selection. Give the rationale for the choice of cases and controls*Cross sectional study*?Give the eligibility criteria, and the sources and methods of selection of participants |
|  |  |  | (*b*) *Cohort study*?For matched studies, give matching criteria and number of exposed and unexposed*Case-control study*?For matched studies, give matching criteria and the number of controls per case |
| Variables | 6 | 7 | Clearly define all outcomes, exposures, predictors, potential confounders, and effect modifiers. Give diagnostic criteria, if applicable |
| Data sources/ measurement | 5-6 | 8* | For each variable of interest, give sources of data and details of methods of assessment (measurement). Describe comparability of assessment methods if there is more than one group |
| Bias | 5-6 | 9 | Describe any efforts to address potential sources of bias |
| Study size | 5 | 10 | Explain how the study size was arrived at |
| Quantitative variables | 6 | 11 | Explain how quantitative variables were handled in the analyses. If applicable, describe which groupings were chosen and why |
| Statistical methods | 7 | 12 | (*a*) Describe all statistical methods, including those used to control for confounding |
|  | 7 |  | (*b*) Describe any methods used to examine subgroups and interactions |
|  | 7 |  | (*c*) Explain how missing data were addressed |
|  | 7 |  | (*d*) *Cohort study*?If applicable, explain how loss to follow-up was addressed*Case-control study*?If applicable, explain how matching of cases and controls was addressed*Cross sectional study*?If applicable, describe analytical methods taking account of sampling strategy |
|  | 7 |  | (*e*) Describe any sensitivity analyses |
| **Results** |  |  |  |
| Participants | 8 | 13* | (*a*) Report numbers of individuals at each stage of study?eg numbers potentially eligible, examined for eligibility, confirmed eligible, included in the study, completing follow-up, and analysed |
|  | 8 |  | (*b*) Give reasons for non-participation at each stage |
|  | 8 |  | (*c*) Consider use of a flow diagram |
| Descriptive data | 8 | 14* | (*a*)Give characteristics of study participants (eg demographic, clinical, social) and information on exposures and potential confounders |
|  | 8 |  | (*b*) Indicate number of participants with missing data for each variable of interest |
|  |  |  | (*c*) *Cohort study*?Summarise follow-up time (eg average and total amount) |
| Outcome data |  | 15* | *Cohort study*?Report numbers of outcome events or summary measures over time |
|  |  |  | *Case-control study?*Report numbers in each exposure category, or summary measures of exposure |
|  | 8 |  | *Cross sectional study?*Report numbers of outcome events or summary measures |
| Main results | 8-9 | 16 | (*a*) Report the numbers of individuals at each stage of the study?eg numbers potentially eligible, examined for eligibility, confirmed eligible, included in the study, completing follow-up, and analysed |
|  | 8-9 |  | (*b*) Give reasons for non-participation at each stage |
|  | 8-9 |  | (*c*) Consider use of a flow diagram |
| Other analyses | 8-9 | 17 | Report other analyses done?eg analyses of subgroups and interactions, and sensitivity analyses |
| **Discussion** |  |  |  |
| Key results | 9 | 18 | Summarise key results with reference to study objectives |
| Limitations | 11 | 19 | Discuss limitations of the study, taking into account sources of potential bias or imprecision. Discuss both direction and magnitude of any potential bias |
| Interpretation | 9-11 | 20 | Give a cautious overall interpretation of results considering objectives, limitations, multiplicity of analyses, results from similar studies, and other relevant evidence |
| Generalisability | 9-12 | 21 | Discuss the generalisability (external validity) of the study results |
| **Other information** |  |  |  |
| Funding | 12 | 22 | Give the source of funding and the role of the funders for the present study and, if applicable, for the original study on which the present article is based |

Supplementary Table 2 Associations between vitamins and carotenoids and liver steatosis and fibrosis in linear regression

|  | Controlled attenuation parameter scores (dB/m) | | | Liver stiffness values (kPa) | | |
| --- | --- | --- | --- | --- | --- | --- |
| Exposure | Model 1 | Model 2 | Model 3 | Model 1 | Model 2 | Model 3 |
| vitamin A,RAE | -0.71 (-1.42-0.01) | -0.56 (-1.23-0.10) | -0.50 (-1.07-0.07) | 0.06 (-0.01-0.14) | 0.07 (-0.00-0.15) | 0.05 (-0.02-0.13) |
| vitamin B1 | -0.55 (-5.31-4.21) | 0.05 (-4.70-4.80) | 0.56 (-3.30-4.43) | 0.19 (-0.03-0.42) | 0.24 (0.02-0.45)* | 0.12 (-0.11-0.35) |
| vitamin B2 | -2.35 (-6.45-1.74) | -2.08 (-6.12-1.95) | -1.87 (-5.03-1.30) | 0.10 (-0.07-0.28) | 0.12 (-0.04-0.29) | 0.03 (-0.11-0.17) |
| vitamin B6 | -0.52 (-3.06-2.02) | -0.48 (-3.00-2.04) | -0.26 (-2.07-1.55) | 0.04 (-0.09-0.17) | 0.05 (-0.08-0.18) | 0.00 (-0.12-0.12) |
| vitamin B12 | 0.20 (-0.90-1.30) | 0.18 (-0.90-1.25) | -0.13 (-0.85-0.59) | 0.08 (0.03-0.12)** | 0.08 (0.03-0.12)** | 0.04 (0.00-0.08)* |
| choline | 0.82 (-1.64-3.28) | 1.33 (-1.09-3.75) | 0.38 (-3.89-4.64) | 0.27 (0.03-0.50)* | 0.29 (0.06-0.51)* | 0.00 (-0.27-0.27) |
| niacin | -0.06 (-0.42-0.31) | -0.04 (-0.40-0.31) | -0.08 (-0.34-0.19) | 0.01 (-0.01-0.02) | 0.01 (-0.01-0.02) | -0.00 (-0.02-0.01) |
| folate, DFE | -0.79 (-1.80-0.23) | 0.60 (-1.63-0.43) | -0.17 (-1.22-0.89) | 0.05 (-0.03-0.13) | 0.06 (-0.02-0.13) | 0.06 (-0.02-0.15) |
| vitamin C | -7.20 (-11.69- -2.70)** | -6.28 (-10.64- -1.92)** | -2.39 (-5.91-1.13) | 0.02 (-0.27-0.31) | 0.06 (-0.21-0.33) | 0.13 (-0.13-0.38) |
| α-carotene | -0.14 (-0.40-0.13) | -0.11 (-0.37-0.16) | -0.05 (-0.24-0.15) | 0.04 (-0.01-0.09) | 0.04 (-0.00-0.09) | 0.04 (-0.00-0.09) |
| β-carotene | -0.12 (-0.21- -0.04)** | -0.10 (-0.18- -0.03)* | -0.08 (-0.14- -0.01)* | 0.00 (-0.01-0.01) | 0.00 (-0.01-0.02) | 0.00 (-0.01-0.02) |
| β-cryptoxanthin | -0.07 (-0.20-0.06) | -0.06 (-0.18-0.05) | -0.01 (-0.09-0.07) | 0.01 (-0.00-0.02) | 0.01 (-0.00-0.02) | 0.01 (-0.00-0.02) |
| serum vitamin C | -19.09 (-24.17- -14.00)** | -18.53 (-23.62- -13.43)** | -5.24 (-9.02 -1.46)** | -1.19 (-1.78- -0.60)** | -1.19 (-1.86- -0.51)** | -0.85 (-1.57- -0.13)* |
| lutein+zeaxanthin | -2.54 (-3.53- -1.54)** | -2.33 (-3.39- -1.26)** | -1.69 (-2.80 -0.58)** | -0.03 (-0.08-0.03) | -0.02 (-0.08-0.04) | -0.01 (-0.06 -0.04) |
| lycopene | -0.28 (-0.58-0.02) | -0.20 (0.50-0.10) | -0.18 (-0.42-0.05) | 0.00 (-0.03-0.03) | 0.00 (-0.03-0.04) | 0.00 (-0.03-0.03) |

Controlled attenuation parameter scores and liver stiffness values with increment of 100 μg/d in vitamin A (RAE), 1 mg/d in vitamin B1, B2 and B6, 1μg/d in vitamin B12, 100 mg/d in choline, 1 mg/d in niacin, 1 μg/d in folate, 10 mg/d in vitamin C, 1 μg/d in alpha-carotene, 100 μg/d in beta-carotene, 10 μg/d in β-cryptoxanthin, 1000 μg/d in lutein+zeaxanthin, 1000 μg/d in lycopene.

T1: tertile 1, T2: tertile 2, T3: tertile 3

*: P<0.05, **: P<0.01

RAE: retinol activity equivalents, DFE: dietary folate equivalents

Model 1 was adjusted for age, sex and race/ethnicity.

Model 2 was adjusted for covariates in model 1, and also education, physical activity, smoking and alcohol drinking.

Model 3 was adjusted for covariates in model 2, and also body mass index, hypertension, diabetes and dietary intakes of cholesterol.

Supplementary Table 3 Associations between vitamins and carotenoids and liver steatosis in sensitivity analysis

|  | Model 1 | | Model 2 | | Model 3 | |
| --- | --- | --- | --- | --- | --- | --- |
| Exposure | T2 vs. T1 | T3 vs. T1 | T2 vs. T1 | T3 vs. T1 | T2 vs. T1 | T3 vs. T1 |
|  | OR (95% CI) | OR (95% CI) | OR (95% CI) | OR (95% CI) | OR (95% CI) | OR (95% CI) |
| vitamin B1 | 1.23 (0.96-1.57) | 1.09 (0.86-1.37) | 1.26 (0.98-1.61) | 1.13 (0.88-1.45) | 1.43 (1.07-1.90)* | 1.21 (0.98-1.49) |
| vitamin B2 | 1.10 (0.85-1.43) | 0.92 (0.66-1.28) | 1.15 (0.88-1.50) | 0.99 (0.71-1.38) | 1.30 (1.02-1.65)* | 1.10 (0.79-1.55) |
| vitamin B6 | 1.00 (0.72-1.40) | 0.93 (0.69-1.24) | 1.01 (0.72-1.42) | 0.97 (0.71-1.33) | 0.99 (0.75-1.32) | 1.00 (0.76-1.32) |
| vitamin B12 | 1.23 (0.87-1.74) | 1.11 (0.83-1.49) | 1.26 (0.91-1.73) | 1.15 (0.86-1.54) | 1.34 (0.99-1.81) | 1.23 (0.90-1.68) |
| choline | 1.08 (0.78-1.49) | 1.05 (0.76-1.47) | 1.10 (0.80-1.51) | 1.13 (0.81-1.58) | 1.17 (0.80-1.71) | 1.21 (0.67-2.20) |
| niacin | 1.18 (0.85-1.64) | 1.06 (0.77-1.46) | 1.22 (0.87-1.72) | 1.12 (0.80-1.56) | 1.26 (0.97-1.65) | 1.12 (0.85-1.47) |
| folate, DFE | 1.19 (0.92-1.55) | 0.92 (0.74-1.15) | 1.23 (0.95-1.58) | 0.97 (0.78-1.21) | 1.24 (0.91-1.68) | 0.99 (0.80-1.23) |
| vitamin C | 0.75 (0.58-0.98)* | 0.65 (0.48-0.87)* | 0.77 (0.59-1.01) | 0.68 (0.51-0.91)* | 0.78 (0.60-1.01) | 0.73 (0.55-0.98)* |
| lutein+zeaxanthin | 1.00 (0.78-1.27) | 0.74 (0.55-1.00) | 1.00 (0.78-1.28) | 0.79 (0.58-1.07) | 0.85 (0.58-1.25) | 0.76 (0.56-1.05) |
| lycopene | 1.06 (0.84-1.34) | 0.97 (0.76-1.22) | 1.07 (0.85-1.36) | 1.00 (0.80-1.25) | 1.27 (0.94-1.70) | 1.02 (0.72-1.44) |

T1: tertile 1, T2: tertile 2, T3: tertile 3

OR (95% CI): odds ratio (95% confidence interval)

*: P<0.05, **: P<0.01

RAE: retinol activity equivalents, DFE: dietary folate equivalents

Model 1 was adjusted for age, sex and race/ethnicity.

Model 2 was adjusted for covariates in model 1, and also education, physical activity, smoking and alcohol drinking.

Model 3 was adjusted for covariates in model 2, and also body mass index, hypertension, diabetes and dietary intakes of cholesterol.

Supplementary Table 4 Associations between vitamins and carotenoids and liver fibrosis in sensitivity analysis

| Exposures | **Model 1** | | **Model 2** | | **Model 3** | |
| --- | --- | --- | --- | --- | --- | --- |
|  | **T2 vs. T1** | **T3 vs. T1** | **T2 vs. T1** | **T3 vs. T1** | **T2 vs. T1** | **T3 vs. T1** |
|  | OR (95% CI) | OR (95% CI) | OR (95% CI) | OR (95% CI) | OR (95% CI) | OR (95% CI) |
| vitamin B1 | 1.33 (0.94-1.89) | 1.03 (0.60-1.76) | 1.36 (0.97-1.89) | 1.09 (0.66-1.79) | 1.34 (0.94-1.89) | 0.95 (0.56-1.62) |
| vitamin B2 | 1.00 (0.70-1.42) | 0.95 (0.66-1.36) | 1.07 (0.76-1.52) | 1.05 (0.76-1.44) | 1.04 (0.76-1.43) | 0.89 (0.63-1.26) |
| vitamin B6 | 1.22 (0.80-1.84) | 1.14 (0.68-1.89) | 1.29 (0.85-1.95) | 1.24 (0.77-1.99) | 1.19 (0.75-1.90) | 1.11 (0.69-1.81) |
| vitamin B12 | 1.33 (0.89-2.00) | 1.28 (0.88-1.87) | 1.38 (0.93-2.05) | 1.36 (0.96-1.92) | 1.30 (0.82-2.05) | 1.13 (0.76-1.68) |
| choline | 1.45 (1.02-2.05)* | 1.42 (1.00-2.01)* | 1.54 (1.11-2.11)* | 1.54 (1.11-2.14)* | 1.38 (1.02-1.86)* | 0.98 (0.62-1.55) |
| niacin | 1.08 (0.69-1.69) | 1.12 (0.68-1.86) | 1.15 (0.75-1.78) | 1.23 (0.78-1.94) | 1.08 (0.64-1.80) | 1.04 (0.63-1.74) |
| folate, DFE | 1.03 (0.66-1.63) | 0.92 (0.53-1.60) | 1.06 (0.69-1.64) | 0.99 (0.60-1.66) | 1.05 (0.67-1.65) | 0.98 (0.57-1.68) |
| vitamin C | 0.78 (0.52-1.18) | 0.88 (0.60-1.30) | 0.82 (0.55-1.23) | 0.96 (0.67-1.40) | 0.85 (0.59-1.24) | 0.99 (0.66-1.49) |
| lutein+zeaxanthin | 1.27 (0.85-1.91) | 1.06 (0.67-1.70) | 1.32 (0.89-1.95) | 1.20 (0.75-1.93) | 1.04 (0.65-1.67) | 1.11 (0.62-1.97) |
| lycopene | 0.72 (0.45-1.15) | 0.86 (0.48-1.53) | 0.73 (0.46-1.16) | 0.89 (0.51-1.57) | 0.78 (0.49-1.25) | 0.91 (0.52-1.59) |

T1: tertile 1, T2: tertile 2, T3: tertile 3

OR (95% CI): odds ratio (95% confidence interval)

*: P<0.05, **: P<0.01

RAE: retinol activity equivalents, DFE: dietary folate equivalents

Model 1 was adjusted for age, sex and race/ethnicity.

Model 2 was adjusted for covariates in model 1, and also education, physical activity, smoking and alcohol drinking.

Model 3 was adjusted for covariates in model 2, and also body mass index, hypertension, diabetes and dietary intakes of cholesterol.
